# Hybrid Immunity Provides the Best COVID-19 Humoral Response in Immunocompromised Patients with or without SARS-CoV-2 Infection History

**DOI:** 10.3390/vaccines11081380

**Published:** 2023-08-18

**Authors:** Paulina Nazaruk, Ignacy Tkaczyk, Marta Monticolo, Anna Maria Jędrzejczak, Natalia Krata, Leszek Pączek, Bartosz Foroncewicz, Krzysztof Mucha

**Affiliations:** 1 Department of Immunology, Transplantology and Internal Diseases, Medical University of Warsaw, 02-006 Warsaw, Poland; tapaulla@wp.pl (P.N.); ignacytkaczyk@gmail.com (I.T.); marta.monticolo@gmail.com (M.M.); anamarie.jedrzejczak@gmail.com (A.M.J.); leszek.paczek@wum.edu.pl (L.P.); bartosz.foroncewicz@wum.edu.pl (B.F.); 2 Department of Clinical Immunology, Medical University of Warsaw, 02-006 Warsaw, Poland; nkrata@wum.edu.pl; 3 ProMix Center (ProteogenOmix in Medicine), Department of Immunology, Transplantology and Internal Diseases, Medical University of Warsaw, 02-006 Warsaw, Poland; 4 Institute of Biochemistry and Biophysics, Polish Academy of Sciences, 02-106 Warsaw, Poland

**Keywords:** COVID-19, SARS-CoV-2, BNT162b2 mRNA vaccine, chronic kidney disease, kidney transplantation, liver transplantation, solid organ transplant, vaccination

## Abstract

Immunization against severe acute respiratory syndrome coronavirus 2 (SARS-CoV-2) has significantly limited the spread of coronavirus disease 2019 (COVID-19) and reduced the associated complications, especially mortality. To prolong immunity, an immune booster was implemented. We evaluated the role of SARS-CoV-2 infection history in the vaccination schedules of kidney and liver transplant recipients and patients with chronic kidney disease (CKD). To this end, we retrospectively analyzed the data of 78 solid organ transplantation (SOT) recipients and 40 patients with immunoglobulin A (IgA) nephropathy as representatives of the CKD group. Patients received two or three doses of the BNT162b2 vaccine. At the follow-up, antibody (Ab) titer, graft function, COVID-19 history, and patients’ clinical condition were assessed. Ab level was higher after two doses in patients with a COVID-19 history over three doses in patients with no COVID-19 history. Compared to three doses, subjects who were administered two doses had a longer median time to infection. Positive antibodies, in response to the third dose, were not observed in up to 8.4% of SOT patients. The results show that the vaccination schedule should take into account the vaccine response rate and COVID-19 history. So-called hybrid immunity appears to be most efficient at providing humoral responses against SARS-CoV-2 infection in immunocompromised patients.

## 1. Introduction

Over the last years, SARS-CoV-2 has spread worldwide and forced us to determine the best strategy against this deadly virus. A key milestone was the development of effective vaccines, which were introduced globally in 2021 [1]. However, it was reported that, by 6 months after vaccination, the efficacy of protection wanes [2,3]. For this reason, administration of the so-called booster vaccination was proposed to prolong antiviral immunity [4]. Hall et al. [3] found that, in the general population, vaccination after previous infection appeared to boost and extend immunity with no signs of waning more than 1 year after primary infection. Additional protection against reinfection was also observed in previously infected individuals [5].

However, these trials did not focus on immunocompromised patients, such as solid organ transplant (SOT) recipients and chronic kidney disease (CKD) patients. The best vaccination strategy, especially in immunocompromised patients, is still under discussion. A weak humoral response in SOT recipients after the first—and even second—dose has been reported [6,7,8,9]; thus, the booster was recommended [10,11,12]. However, despite three doses of the mRNA vaccine, nearly 50% of SOT recipients did not develop the expected immunological response [13]. Our group previously reported different post-vaccination responses in kidney and liver transplant recipients (KTRs 57.1% vs. LTRs 88.9%) [14]. Taking all of these factors into consideration, some countries have recommended a fourth vaccine dose, even though the effectiveness of such a strategy is disputable [15,16]. A major counterargument is the potential side effects of a subsequent booster, particularly in immunocompromised patients.

The potential role of infection-acquired immunity in SOT recipients was reported by Boyarsky et al. [17], who showed that convalescent SOT vaccines have a higher post-dose antibody titer (anti-S1Ab) than naive vaccines. In addition, another study showed that efficacy might be sufficient in KTRs with detectable S1/S2 immunoglobulin Gs (IgGs) before the BioNTech/Pfizer COVID-19 mRNA (BNT162b2) vaccination [18].

Patients with CKD are another group with an increased risk of severe disease and COVID-19-related death, even after two doses of BNT162b2 [19]. Furthermore, in CKD patients who are not treated with immunosuppressants (IS), the efficacy of vaccines might be hampered [20]. To date, there have been no long-term anti-SARS-CoV-2 vaccination efficacy trials in patients with non-dialysis-dependent CKD.

The aim of our study was to compare the efficacy of the anti-SARS-CoV-2 vaccination scheme in SOT and CKD patients with and without a COVID-19 history.

## 2. Materials and Methods

### 2.1. Study Design

**Figure 1:**
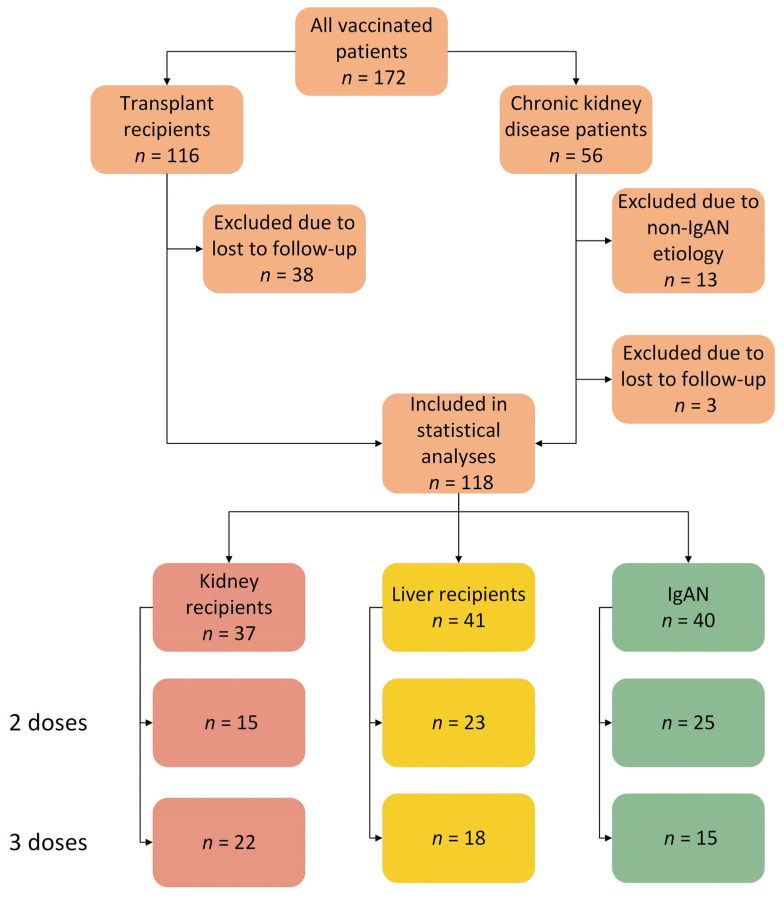
Study group flow chart. IgAN = immunoglobulin A nephropathy.

We retrospectively analyzed the data of 172 patients: 116 SOT recipients (61 KTRs and 55 LTRs) and 56 CKD patients who were not taking multiple IS and served as a control group for SOT. In total, 37 KTRs and 41 LTRs who had anti-S1Ab measurements for 6 months after the second vaccine dose were included in the subsequent analyses. As representatives of CKD, we enrolled 40 patients with IgA nephropathy (IgAN) who had follow-ups after vaccination (Figure 1).

The mean participant ages were 53.1 years (KTRs), 52.8 years (LTRs), and 50.1 years (IgAN). The study group consisted of 72 (61%) female and 46 (39%) male participants.

Depending on the type of transplanted organ, SOT patients received various combinations of immunosuppressive maintenance treatments. Mono-, dual-, or triple-drug regimens were based on azathioprine (AZA), cyclosporine (CsA), everolimus (EVR), glucocorticosteroids (GCs), mycophenolate mofetil (MMF) or sodium (MPA), sirolimus (SIR), and tacrolimus (TAC). To enable comparison among different IS protocols, the daily IS dose was converted to AU (1 AU is equal to 1 mg TAC, 100 mg CsA, 1 mg SIR, 1 mg EVR, 1000 mg MMF, 720 mg MPA, 50 mg AZA, 5 mg prednisone, and 6.25 mg methylprednisolone).

All study participants received two or three 30 μg doses of BNT162b2 vaccine between 13 January 2021 and 8 March 2022. Their follow-ups were performed during routine outpatient visits between doses and after full vaccination at consecutive time points (TPs): TP1, 4–6 weeks after the first dose; TP2, 4–8 weeks after the second dose; TP3, 9–20 weeks after the second dose; TP4, 21–32 weeks after the second and 1–12 weeks after the third dose; TP5, 33–48 weeks after the second and 4–20 weeks after the third dose. Patients’ clinical statuses, anti-S1 Ab serum levels, and selected biochemical parameters were assessed. All patients had stable graft function without signs of chronic disease exacerbation or a COVID-19 history for 2 months prior to vaccination.

COVID-19 presence was diagnosed with a positive PCR test. Antigen test or COVID-like symptoms described in medical interviews were not sufficient for diagnosis. Patients with positive PCR test results were asked about the severity of infection, including questions regarding the necessity of hospitalization, muscle pain, cough, sore throat, fever, shortness of breath, tachycardia, loss of taste and/or smell, as well as diarrhea.

### 2.2. Anti-S1Ab Testing

The methods used for anti-S1Ab evaluation were published previously [14]. The SARS-CoV-2 IgG II Quant assay (Abbott Laboratories, Chicago, IL, USA) was used. Formerly, results were presented in arbitrary units per mL (AU/mL). After July 2021, the hospital’s laboratory reported results in binding Ab units per mL (BAU/mL), following the World Health Organization standards [21]. For the analytical measuring interval ranges from 3.0 to 5680 BAU/mL, the cut-off is 7.1 BAU/mL, results ≤7.1 BAU/mL are considered negative, and >7.1 BAU/mL are positive. The AU/mL results were converted into BAU/mL according to [22], as below:

BAU/mL = 0.142 × AU/mL

We also present the difference between the anti-S1Ab titer at TP3 and TP5 after the second dose as delta (Δ) anti-S1Ab.

### 2.3. Biochemical and Clinical Tests

We assessed basic biochemical parameters, such as alanine aminotransferase (ALT), alkaline phosphatase (ALP), aspartate aminotransferase (AST), bilirubin, hemoglobin, gamma-glutamyl transpeptidase (GGTP), serum creatinine, and estimated glomerular filtration rate (eGFR). Tests were performed with automatic analyzers: Cobas Integra 400 Plus and Elecsys 2010 (Roche Diagnostics, Mannheim, Germany). To estimate the eGFR, we used the CKD Epidemiology Collaboration creatinine equation. In addition, we also evaluated the body mass index (BMI).

### 2.4. Statistical Analyses

For statistical analyses, R version 3.6.1, Statistica version 13.3 (StatSoft, Tulsa, OK, USA), and GraphPad Prism 9.3 (GraphPad software, San Diego, CA, USA) were used. Results are presented as percentage value, median (MD) ± interquartile range, or the mean ± standard deviation (SD). Chi-square test was used for the evaluation of statistical differences between selected variants of answers and groups. Non-parametric tests—the Mann–Whitney U test and Kruskal–Wallis test—were used to analyze variables with non-normal distribution. The normal distribution of the variables was checked by the Shapiro–Wilk test. Spearman’s rank correlation allowed for the verification of the relationship between the parameters. *p* values below 0.05 were considered significant.

### 2.5. Approval

This study was approved by the institutional review board of the Medical University of Warsaw (AKBE/100/2023; Warszawa, Poland).

## 3. Results

### 3.1. Comparison of the Analyzed Groups

Among all the study participants, there were a significant differences in age (*p* = 0.025), BMI (*p* = 0.013), and creatinine (*p* = 0.026). Other clinical parameters remain non-significant.

There were 52 (67%) SOT recipients on TAC therapy, and 24 (31%) were on CsA. Statistical differences were found in relation to the type of immunosuppressive drugs received by the study groups: GCs, MMF, CsA (*p* < 0.001), and TAC (*p* = 0.002). The main IS schemes were triple therapy in KTRs (73%), double therapy in LTRs (43%), and monotherapy in IgAN patients (22.5%). The largest IS load was taken by the KTRs (4.8 AU; *p* < 0.001). Additionally, a positive history of COVID-19 was found in 19 patients, only 3 of whom required hospitalization. Fever was the most common symptom. The baseline characteristics of the patients are presented in Table 1.

### 3.2. Immune Response

The vaccination interval ranged from 3 to 6 weeks between the first and second doses in all study groups, whereas it ranged from 19 to 45 weeks (MD = 30 weeks) between the second and third doses in SOT recipients and 13 to 48 weeks (MD = 30 weeks) in IgAN patients. The distribution of patients who received an immune booster, namely those who had a prior infection or received a third vaccine dose, are summarized in Table 2.

**Figure 2:**
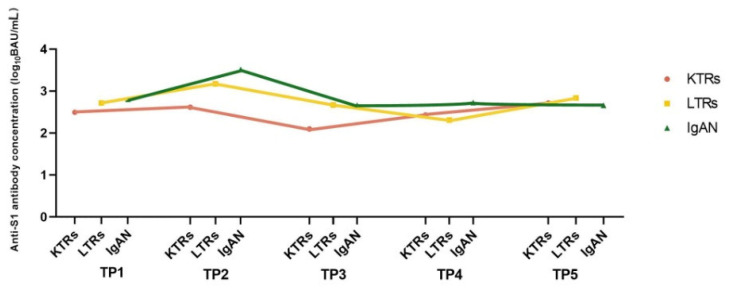
Anti-SARS-CoV-2 spike protein S1Ab concentration (BAU/mL) after BNT162b2 vaccine in KTRs, LTRs, and IgAN patients at five time points (TPs). Values are presented as a linear graph with median expressed in log_10._

We focused on the observation of fluctuations of anti-S1Ab serum levels from 9 to 48 weeks after the second vaccine dose, comparing them to the early period after the second dose. Detailed results of the first 8 weeks (TP1–2) after the second dose have been published [14]. The Ab concentrations during the entire observation period in KTRs, LTRs, and IgAN are presented in Figure 2.

The differences in Ab titers among the LTRs, KTRs, and IgAN patients at each TP were non-significant. An average of a 62.8% decrease in TP3 Ab titer was observed in all study groups compared to TP2 (Table 3). The highest decrease by 68.4% at TP3 was recorded in the KTRs.

There were 13 patients (11%) who no longer had a positive serum level of anti-S1Ab (Ab titer below ≤7.1 BAU/mL) at TP3. These patients also had a low immune response up to 8 weeks after the second vaccine dose, which was typically below the cut-off value. This group included SOT patients only, and most were KTRs (*n* = 9). Ab titer did not significantly differ among groups at TP3 (*p* = 0.144). Throughout the follow-up period, 55 patients received a third vaccine dose, mostly at TP4 (Table 2). At this time, as well as at TP2, SOT patients had distinctly lower levels of Ab compared to IgAN patients without statistical significance (*p =* 0.05; Table 4). In addition, the KTRs had lower Ab titers than the LTRs.

After the third vaccine dose, a gradual increase in Ab titer was observed at TP4 and TP5, but it was not as high as that at TP2 after the second dose (Table 4 and Table 5). We found statistically significant differences among the groups at TP4 after the third dose was received at TP3 (*p* = 0.002). The post hoc analysis with multiple pairwise comparisons was performed, obtaining the following results: KTRs vs. IgAN *p* = 0.022, LTRs vs. IgAN *p* = n.s, KTRs vs. LTRs *p* = n.s.

Table 5 shows an increase in Ab levels to peak values at TP5 in LTRs who received the third vaccine dose at TP3, possibly because they had asymptomatic virus infections.

The positive antibody response after the third vaccine dose was not observed in 10 (8.4%) patients (7 KTRs and 3 LTRs) at TP4. Most of them did not achieve a positive level of anti-S1Ab during the entire follow-up. For comparison, in the two-dose regimen, only two patients did not have a positive response at TP4. We identified a group of four KTRs (3.4%) and two LTRs (1.7%) who did not achieve a positive Ab level at TP5 despite three doses of vaccine. None of them had a COVID-19 history in our follow-up, indicating the potential role of other lines of defense in the immune system, such as T cell response or the IS dose. Immunocompromised patients were more likely to follow a hand sanitizing regime and social distancing recommendations, which could also matter [23]. Interestingly, the mean daily IS dose in these patients was 3.25 AU, which was higher than the average for the entire study group (3.03 AU). There were five patients in the three-dose scheme who did not respond to two doses of vaccination (Ab titer <7.1 BAU/mL) at TP3 and TP4, but they achieved a positive antibody response at TP5. This could be either a result of the third vaccine or, more than likely, asymptomatic virus infection. This suggests that the natural booster is more effective.

**Figure 3:**
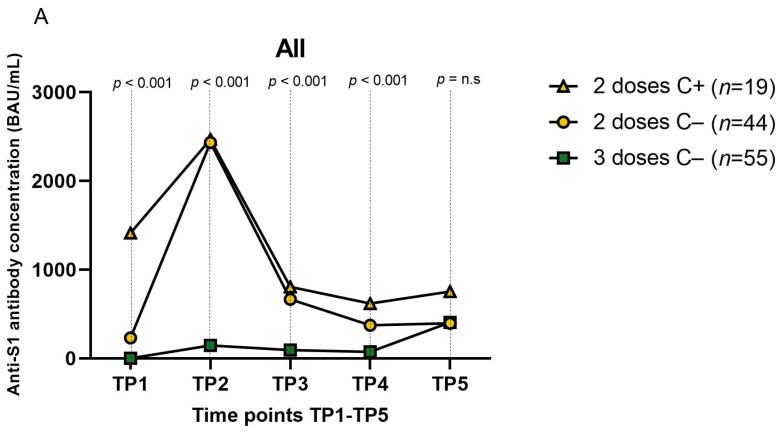

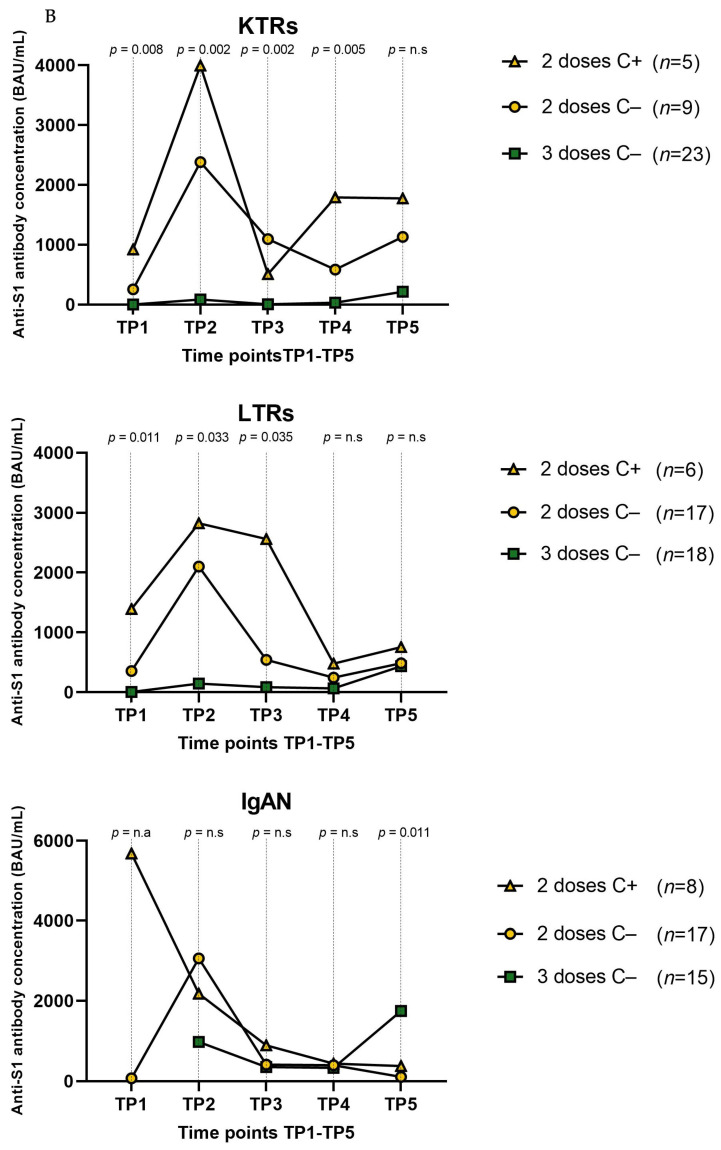
Anti-SARS-CoV-2 spike protein Ab concentration (BAU/mL) after BNT162b2 vaccination in the immunization groups depending on the number of vaccination doses and COVID-19 history confirmed by a positive PCR test. (**A**) The relationship between the schemes of the received immune boosters and linear graph with median for all participants at TP1–5; values are set as the median (MD); the *p*-value was calculated with the non-parametric Kruskal–Wallis test, and *p* < 0.05 was considered statistically significant. (**B**) A comparison of immunization groups in KTRs, LTRs, IgAN at TP1–5; values are presented as a linear graph with the median; the p-value was calculated with the non-parametric Kruskal–Wallis test. C+ = positive SARS-CoV-2 PCR test; C− = negative SARS-CoV-2 PCR test; *n* = number of observations; n.a. = not available; n.s. = not significant. For detailed analysis, please see the Appendix section, Table A1 and Table A3.

Considering the above, we analyzed Ab titers in patients who received an immune booster with the third vaccine dose or with infection. Objects who had a positive SARS-CoV-2 PCR test were designated C+, and those without a COVID-19 history were C−. We divided the C+ and C− groups based on the number of vaccine doses received. Finally, we identified three main immunization groups: two-dose C+, two-dose C−, and three-dose C− (Figure 3A); there were no participants with three vaccine doses and C+ designation. Significant relationships were found at TP1–4 between the Ab titer in the two-dose C− and three-dose C− groups (*p* < 0.001–0.005) and between the two-dose C+ and three-dose C− groups (*p* < 0.001–0.005). Delta S 3–5 was significantly different between the two-dose C− and three-dose C− groups (*p* < 0.001). Significant correlation between Ab titer in the two-dose C+ and two-dose C− groups was found only at TP1 (*p* = 0.042). There was no significant relationship of Ab titer in the immunization groups at TP5. Further analyses of the subgroups of the KTRs, LTRs, and IgAN patients revealed a relationship among the three immunization groups in the KTRs at TP1–4 (*p* = 0.002–0.008), LTRs at TP1–3 (*p* = 0.011–0.035), and IgAN patients at TP5 (*p* = 0.011) (Figure 3B; Appendix, Table A1).

**Figure 4:**
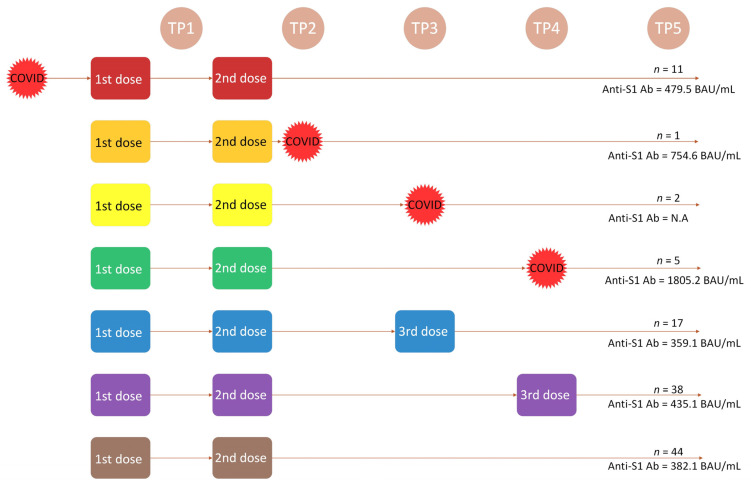
Main immunization tracks. *n* = number of observations; values are set as median (MD) at TP5. Anti-S1Ab = SARS-CoV-2 spike protein antibody in BAU/mL; COVID = SARS-CoV-2 infection; 1st, 2nd, 3rd dose = doses of BNT162b2 vaccine.

Analyzing the above four schemes, we referred them to the vaccination calendar and detailed the above division by placing each immunization (vaccination dose and infection) on the time axis according to the established TPs. We identified seven main immunization tracks (Figure 4).

The diagram presents the median Ab titer at TP5. Ab values were higher in the infection patterns. This was clearly seen in the example of the green and purple tracks. Detailed data and comparison of Ab values in each track at all TPs are presented in Table 6. The order of group designation chronologically corresponds with the immunization scheme that each participant received. In the two-dose scheme, we separated five groups: four of them were C+, and one group was C− (two-dose C−). TP in the header, after the C+ mark, refers to the time when the infection occurred (two-dose C+ TP2, two-dose C+ TP3, two-dose C+ TP4). The notation two-dose C+ means infection prior to vaccination. In the three-dose scheme, there were two groups designated C−. TP indicates the time the third dose was received (three-dose C− TP3; three-dose C− TP4).

Ab titer at different TPs were compared among the schemes. Statistical significance was demonstrated among subgroup Ab at TP1–4 and delta S 3–5 (*p* = < 0.001–0.001). Among 52 patients, 8 (15%) had COVID-19 infections at 31.5 weeks (MD) after the second vaccine dose. All patients had a positive Ab titer before the disease occurred. However, without the three-dose booster or natural infection (two-dose C−), the delta S 3–5 was negative and the Ab level steadily declined. The 11 patients who became ill before the vaccination schedule (two-dose C+) showed a steady decline in Ab titer over time. However, they had relatively high Ab levels at TP3–4 compared to the other schedules, and they were not re-infected during the follow-up period.

In the three-dose schedule, patients vaccinated at TP3 produced higher Ab titer than those who received a third dose at TP4. None of these groups became infected during follow-up.

Patients in the three-dose schedule had lower absolute Ab titer at TP3–4 than those in the two-dose C− schedule (median at TP3: 83.2–111.8 vs. 665 BAU/mL), and they were often below the cut-off value. This was particularly evident in the three-dose C− TP4 group, where the statistical significance ranged from *p* < 0.001 to *p* = 0.002 at TP1-4 (Appendix, Table A2). This is likely why they originally qualified for the third vaccine dose.

Patients who were ill prior to the vaccination course (two-dose C+) also produced a higher Ab titer after vaccination with the second compared to the third dose. There is a relationship with the C− TP4, and statistical significance was shown at TP1–4 in the range of *p* = 0.001–0.009. Scheme two-dose C+ also had higher Ab levels at TP1–3 (*p* = 0.003–0.01) than the three doses C− TP3 scheme (Appendix, Table A2).

The absolute values were also lower in the two-doses C− scheme compared with the two-dose C+ scheme, and statistical significance was only found in TP1-2 *(p* = 0.003–0.04).

No statistical significance was observed in the immunization patterns among the KTRs, LTRs, and IgAN patients in TP5.

In the process of collecting additional information, we found that 8 more participants had a positive SARS-CoV-2 PCR test (3 KTRs, 2 LTRs, 3 IgAN patients) after the observation period. Among them, 2 (1 KTR and 1 LTR) in the two-dose schedule were infected 39 weeks (MD) after the second vaccine dose. The fact that the KTR was free of SARS-CoV-2 infection for 10 months after vaccination, even though his Ab levels were 0.0 BAU/mL during follow-up, can be regarded as a unique phenomenon. The remaining 6 subjects in the three-dose scheme were C+ at 9 weeks (MD) after the third vaccine dose. Their Ab titer at TP3 was <200 BAU/mL.

There was no statistically significant association between the immune response (in all groups and at all TPs) and organ function, daily dose of IS, time from transplantation, nephropathy diagnosis, age, sex, BMI, or blood group.

## 4. Discussion

We found that the number, time, and type of booster influenced the SARS-CoV-2 humoral response in immunocompromised patients. Patients with a history of COVID-19 infection after two doses of BNT162b2 vaccine had higher anti-S1Ab levels at TP1–4 than those who received three doses without the disease with a delayed third dose. They also had higher Ab levels at TP1–3 than recipients of three doses administered in the standard scheme. Thus, prior infection may have led to greater antiviral response in our patients. Infection-acquired immunity combined with vaccination is reportedly long-lasting in the general population [3], and higher anti-S1 Ab levels in previously infected SOT recipients have been reported [17,18]. Earlier infection effectiveness against omicron BA.4 and BA.5 has also been observed [24]. Our study shows that hybrid immunization seems to provide the best humoral response for immunocompromised subjects, which is in line with recent findings in the general population. Hybrid immunity conferred the strongest protection against BA.1 and BA.2 omicron subvariants in the whole vaccinated Qatari population [25]. The hybrid model is also valuable for cross-variant virus neutralizing [26]. For comparison of previous infection after three doses, immunocompetent recipients with the two dose-and-infection scheme would be valuable for further discussion on boosters.

Furthermore, recipients of three doses had lower median anti-S1Ab levels at TP3 (after the second dose) than those who received two vaccine doses. The lower median Ab level at TP3 was likely related to the three vaccine doses received by this group, and it resulted in the maintenance of higher Ab levels at TP5 than the two-dose regimen. Similar indications and findings have been observed in SOT recipients who received a third dose of the mRNA-1273 vaccine [27]. These results may support the arguments against further boosters in immunocompetent patients [13,15,16]. More importantly, they also prove the efficacy of measuring Ab levels to identify the best individualized vaccination algorithm. It has been reported that an individualized vaccination scheme is crucial for SOT recipients [28]. The intergroup variability in Ab serum concentration may be caused by multiple factors, such as genetics, type of transplanted organ, and the total load of immunosuppression [9]. Most of our patients did not develop symptomatic COVID-19 during the vaccination process. However, 8 of 52 (15%) two-dose recipients developed COVID-19 despite having positive Ab levels at a preceding time point. Clearly, Ab formation does not prevent viral invasion, but it is expected to reduce symptoms and shorten the disease duration. Of note, neutralization escape by some SARS-CoV-2 subvariants related to various mutations in the spike protein has been identified [29]. Multiple mutations elsewhere in the virus, resulting in new variants, may also matter [30]. Of note, we used the term positive humoral response based on the binding activity of the antibodies. We are aware that additional neutralizing experiments with viral isolates or a pseudovirus would be needed to prove if positive humoral response is also protective against COVID-19 [31,32,33,34]. However, taking into account the clinical practice and wide use of antibody titer as a biomarker to monitor the response to vaccination, the terms positive response and protective response are frequently used as synonyms.

The need to adjust vaccination schemes in immunocompromised patients because of inadequate immune response emerged [35]. To the best of our knowledge, there are no data concerning immunocompromised patients in as many vaccination schemes as presented in this study. We assigned study participants to seven different subgroups, following four vaccination schemes depending on the number of vaccine doses, vaccination intervals, and time of previous infection. Such division may be relevant in identifying the best vaccination approach for immunocompromised patients. As aforementioned, a personalized vaccination scheme seems to be most desirable in immunocompromised patients; however, dividing individuals into so many groups could also limit the statistical power. Furthermore, immunocompromised patients are a non-homogenous group, including not only solid organ transplant recipients (SOT) but also other patients, such as people with human immunodeficiency virus (HIV) infection, cancers, and primary immunodeficiencies. The underlying etiology results in different seroconversion rates in this group [36]. In regards to this, finding the best vaccination scheme in other immunocompromised patients is needed.

During follow-up, we assessed anti-S1Ab titer levels at five different TPs (TP1–5) depending on the time of immunization. The longest follow-up time was 4–20 weeks after the third dose (TP5). Taking into consideration waning immunity, especially in immunocompromised patients, prolonged observation enabled us to find the most durable vaccination scheme. However, further observation is needed to assess antibody response in every given group and vaccination scheme. It is necessary to be aware that the withdrawal of population-wide PCR testing can hamper the knowledge of future possible infection-acquired immunity. New virus variants may additionally boost humoral response and influence immunity duration and vaccination scheme selection. Long-term observations will be crucial for identifying the most effective strategy.

This study had some limitations. It did not include patients who received three vaccine doses and were infected during their follow-ups, and the subgroup sizes were relatively small. Additionally, only using the PCR test to confirm SARS-CoV-2 infection may have biased our analyses, and we realize that some patients became infected after our follow-up. Moreover, some patients had COVID-like symptoms but did not test themselves or only performed the antigen test, which excluded them from qualifying for the group that was previously infected. We investigated the humoral response indicated by both vaccines and infection; however, T-cell response is also crucial for providing protection [37]. Information about T-cell responses would better describe immunological response [38]. The retrospective study design precluded cellular response evaluation. Lastly, we did not compare immunocompromised patients with healthy controls. Nevertheless, an immunological response in healthy subjects was established [39,40,41,42,43,44].

## 5. Conclusions

Taking into account our results and the available literature, vaccination schemes should be reevaluated with regard to COVID history. Immunocompromised patients are a non-homogeneous group that may differ in their immune response to vaccines. Our results provide a rationale for personalizing the vaccination scheme based on factors such as serum Ab levels, medical history, and IS dose. We suggest that hybrid immunity might provide the best humoral response; however, this needs to be confirmed in both the general population and immunocompromised patients.

## Figures and Tables

**Table 1:** Demographic and clinical characteristics of the study participants.

Characteristics	KTRs *n* = 37	LTRs *n* = 41	IgAN *n* = 40	*p*-Value
Mean age (SD), years	53.1 (13.4)	58.2 (12.6)	50.1 (13.8)	^†^ 0.025
Median age (range), years				
All	53 (18–77)	61 (26–77)	50 (23–78)	
Male	51 (18–71)	61 (47–70)	47 (23–71)	
Female	55.5 (31–77)	61 (26–77)	50 (23–78)	
Sex (%), *n*				
Male	17 (45.9)	10 (24.4)	19 (47.5)	* n.s
Female	20 (54.1)	31 (75.6)	21 (52.5)	
Mean BMI (SD), kg/m^2^	24.8 (4.1)	25.7 (4)	28 (4.7)	^†^ 0.013
Mean time since transplantation (SD), years	13.4 (6.5)	14.8 (3.9)	n.a	^††^ n.s
Mean time since biopsy (SD), years	n.a	n.a	17.8 (10.8)	n.a
COVID-19				
(confirmed by PCR), *n* (%)	5 (13.5)	6 (14.6)	8 (20)	* n.s
Hospitalization due to COVID-19 **	2 (25)	1 (12.5)	0	* n.s
Mean saturation, %	98	94.2	96	^†^ n.s
Symptoms **				
Fever >38 °C	5 (62.5)	6 (100)	4 (50)	* n.s
Loss of smell and/or taste	0	4 (66.7)	4 (50)	* n.s
Dyspnea	1 (20)	2 (33.3)	0	n.a
Sore throat	2 (40)	5 (83.3)	3 (37.5)	* n.s
Myalgia	2 (40)	6 (100)	4 (50)	* n.s
Cough	3 (60)	6 (100)	5 (62.5)	* n.s
Pneumonia	1 (20)	1 (16.7)	0	* n.s
Diarrhea	1 (20)	5 (83.3)	0	* n.s
Tachycardia/arrythmia	0	4 (66.7)	0	n.a
Other	2 (40)	2 (33.3)	3 (37.5)	* n.s
Therapy during COVID infection				
Steroids	0	0	1 (12.5)	n.a
Antibiotics	3 (60)	3 (50)	0	* n.s
Remdesivir	0	0	0	n.a
Immunosuppression, *n* (%)				
Steroids	33 (89.2)	16 (39)	11 (27.5)	* <0.001
Mycophenolate mofetil	26 (70.3)	10 (24.4)	0	* <0.001
Azathioprine	5 (13.5)	4 (9.8)	0	* n.s
Cyclosporine	16 (43.2)	8 (19.5)	2 (5)	* <0.001
Tacrolimus	20 (54.1)	32 (78)	0	* 0.002
Sirolimus	0	1 (2.4)	0	n.a
Everolimus	1 (2.7)	0	0	n.a
Mean daily dose, AU (SD)	4.8 (2.7)	3.1 (1.9)	1.2 (1)	* <0.001
Immunosuppression scheme, *n* (%)				
Monotherapy (CNI/MMF/GCs)	0	18 (43.9)	9 (22.5)	* 0.013
Dual therapy (CNI + GCs/MMF/AZA/mTORi)	10 (27)	16 (39)	2 (5)	* 0.001
Triple therapy (CNI/mTORi + GCs + MMF/AZA)	27 (73)	7 (17.1)	0	* <0.001
Mean laboratory data (SD)				
ALT, IU/L	22.3 (11.9)	33.1 (63.3)	n.a	^††^ n.s
AST, IU/L	25 (3.5)	31.8 (46.2)	n.a	^††^ n.s
GGTP, IU/L	n.a	52.6 (48.9)	n.a	n.a
ALP, IU/L	n.a	107.1 (53)	n.a	n.a
Bilirubin, mg/dL	n.a	0.7 (0.5)	n.a	n.a
Hemoglobin	13.6 (2)	13.3 (1.4)	13.6 (1.9)	^†^ n.s
Serum creatinine, mg/dL	1.4 (0.9)	1 (0.2)	1.8 (1.2)	^†^ 0.026
eGFR, mL/min * 1.73 m^2^	61.4 (27.7)	72.8 (17.3)	57.9 (41.3)	^†^ n.s

Parameters evaluated at (TP5), ** = % of infection confirmed by PCR. CNI = calcineurin inhibitor including cyclosporine and tacrolimus; mTORi = mammalian target of rapamycin kinase inhibitor including everolimus and sirolimus. The *p*-value was calculated with the non-parametric Kruskal–Wallis test (**^†^**) and Mann–Whitney U test (**^††^**) or Chi-square test (*). *p* < 0.05 was considered statistically significant, n.a = not available, n.s = not significant.

**Table 2:** The number of patients who received a third dose of BNT162b2 vaccine and had a positive history of COVID-19 at five time points.

	0 *	TP1	TP2	TP3	TP4	TP5	
*n*							Total
3rd dose received	0	0	0	17	38	0	55
Positive PCR test ^†^	11	0	1	2	5	0	19

* period before the first dose. ^†^ Severe acute respiratory syndrome coronavirus 2 (SARS-CoV-2)-positive PCR test. *n* = number of subjects.

**Table 3:** Decreases in SARS-CoV-2 anti-S1Ab after the second dose of BNT162b2 at TP3 compared to TP2.

Parameter	KTRs	LTRs	IgAN	All
Ab titer decrease, %	68.4	62.8	49.9	62.8

Ab—antibody, TP—time point.

**Table 4:** Comparison of SARS-CoV-2 S1Ab concentration (BAU/mL) after BNT162b2 vaccine in KTRs, LTRs, and IgAN patients.

	KTRs (*n* = 37)	LTRs (*n* = 41)	IgAN (*n* = 40)	
		MD (Q1 to Q3)		*p* value
TP1	10.8 (0.0 to 758.9)	59.0 (3.5 to 1477.5)	2875.3 (1473.0 to 4277.6)	n.s
TP2	428.3 (7.7 to 1747.8)	1130.2 (113.9 to 3322.4)	2451.9 (1137.6 to 3610.0)	n.s
TP3	104.4 (4.4 to 854.2)	330.3 (67.7 to 991.8)	364.3 (224.4 to 770.7)	n.s
TP4	184.4 (21.5 to 668.1)	166.6 (32.6 to 933.6)	419.0 (126.4 to 2060.3)	n.s
TP5	408.8 (121.2 to 1863.9)	535.9 (252.9 to 1941.2)	376.7 (217.2 to 1103.4)	n.s
Delta S 3-5	83.4 (−217.6 to 1207.1)	4.7 (−322.2 to 519.4)	−577.6 (−831.0 to −324.1)	n.s

*n* = number of observations; values are set as the median (MD) and quartile interval (QR1–QR3). The *p*-value was calculated with the non-parametric Kruskal–Wallis test, and *p* < 0.05 was considered statistically significant (comparison of variables among the KTRs, LTRs, and IgAN patents). Delta S 3–5 = the difference in anti-S1Ab titer between TP3 and TP5 after the second dose; n.s = not significant.

**Table 5:** SARS-CoV-2 anti-S1 Ab titer at TP4 and TP5 after the third dose of BNT162b2 vaccine.

		KTRs		LTRs		IgAN		*p* value
MD (Q1–Q3)								
3rd dose received at TP3	TP4	*n* = 7	223.4 (69.3 to 315.7)	*n* = 4	2564.8 (2152.4 to 2600.9)	*n* = 6	2488.2 (1666.5 to 3472.7)	0.002
	TP5		235.9 (104.1 to 395.9)		5680.0 (5680.0 to 5680.0)		n.a	n.a
3rd dose received at TP4	TP4	*n* = 15	249.4 (42.4 to 1576.5)	*n* = 14	31.6 (15.0 to 92.1)	*n* = 9	142.2 (70.5 to 171.6)	n.s
	TP5		716.5 (119.8 to 5300.8)		421.1 (33.4 to 985.0)		1745.5 (1317.5 to 2797.6)	n.s

*n* = number of observations; values are set as median (MD) and quartile interval (QR1–QR3); the *p*-value was calculated with the non-parametric Kruskal–Wallis test, and *p* < 0.05 was considered statistically significant (comparison of variables among KTRs, LTRs, and IgAN patients), n.a = not available; n.s = not significant.

**Table 6:** SARS-CoV-2 anti-S1Ab titer at TP1–5, according to the number of BNT162b2 vaccine doses and COVID-19 history.

SubGroups	2-Dose C+ (*n* = 11)	2-Dose C + TP2 (*n* = 1)	2-Dose C + TP3 (*n* = 2)	2-Dose C + TP4 (*n* = 5)	3-Dose C−TP3 (*n* = 17)	3-Dose C−TP4 (*n* = 38)	2-Dose C− (*n* = 44)	*p* Value
MD								
TP1	2965.9 (2413.7 to 5438.7)	511.8 (511.8 to 511.8)	8.9 (8.9 to 8.9)	875.6 (440.6 to 877.4)	22.3 (0.0 to 246.5)	1.7 (0.0 to 10.4)	220.3 (18.3 to 1371.8)	0.001
TP2	5231.7 (3995.4 to 5680.0)	4517.6 (4517.6 to 4517.6)	721.0 (721.0 to 721.0)	1284.8 (884.8 to 1551.6)	122.1 (6.8 to 613.6)	150.4 (15.6 to 595.6)	2312.5 (1032.2 to 4422.5)	<0.001
TP3	2062.4 (1409.6 to 3332.0)	n.a	365.9 (295.1 to 436.6)	538.6 (474.2 to 603.0)	111.8 (5.2 to 281.6)	83.2 (3.8 to 215.6)	665.5 (255.9 to 1703.0)	<0.001
TP4	727.5 (619.3 to 2353.3)	n.a	5680.0 (5680.0 to 5680.0)	134.1 (74.1 to 281.9)	485.1 (262.4 to 2564.8)	32.0 (5.3 to 123.5)	368.9 (154.1 to 1338.0)	<0.001
TP5	479.5 (441.4 to 1892.8)	754.6 (754.6 to 754.6)	n.a	1805.2 (1241.2 to 3742.6)	359.1 (106.9 to 3044.1)	435.1 (34.1 to 1581.6)	382.1 (200.5 to 2852.6)	n.s
Delta S 3-5	−1084.5 (−2334.6 to −800.8)	n.a	n.a	702.6 (356.1 to 1049.0)	107.1 (83.4 to 5587.0)	311.9 (24.0 to 1730.9)	−316.0 (−538.2 to −130.5)	<0.001

*n* = number of observations; values are set as median (MD) and quartile interval (QR1–QR3); the *p*-value was calculated with the non-parametric Mann–Whitney U test. *p* < 0.05 was considered statistically significant. Subgroup markings were introduced chronologically: 2-doses C+ TP2 = subject obtained two doses of vaccine and then had SARS-CoV-2 infection at TP2; 2-dose C+ TP3 = subject obtained two doses of vaccine and then had SARS-CoV-2 infection at TP3; 2-dose C+ TP4 = subject obtained two doses of vaccine and then had SARS-CoV-2 infection at TP4; 3-dose C− TP3 = subject obtained subject obtained three doses of vaccine, with the third dose at TP3, and did not have previous SARS-CoV-2 infection; 3-dose C− TP4 = subject obtained three doses of vaccine, with the third dose at TP4, and did not have previous SARS-CoV-2 infection; 2-dose C+ = subject had SARS-CoV-2 infection before the two-dose scheme; 2-dose C− = subject obtained two doses of vaccine and did not have previous SARS-CoV-2 infection. Delta S 3–5 = difference between anti-S1Ab titer at TP3 and TP5 after the second dose. C+ = positive SARS-CoV-2 PCR test; C− = negative SARS-CoV-2 PCR test; n.a = not available; n.s = not significant. For details see Appendix, Table A2.

## Data Availability

The data presented in this study are available under reasonable request from the corresponding author.

## Appendix A

**Table A1:** Comparison of Anti-SARS-CoV-2 spike protein Ab concentration (BAU/mL) after BNT162b2 vaccination in the immunization schemes, depending on the number of vaccination doses and COVID-19 history presented by *p* value.

Scheme	2-Dose C−	2-Dose C+	3-Dose C−	2-Dose C−	2-Dose C+	3-Dose C−	2-Dose C−	2-Dose C+	3-Dose C−
**KTRs**				**LTRs**			**IgAN**		
TP1									
**2-dose C−**	x			x			x		
**2-dose C+**	n.s	x		n.s	x		n.a	x	
**3-dose C−**	n.s	0.035	x	0.027	0.027	x	n.a	n.a	x
TP2									
**2-dose C−**	x			x			x		
**2-dose C+**	n.s	x		n.s	x		n.a	X	
**3-dose C−**	0.026	0.005	X	n.s	0.049	x	n.a	n.a	x
TP3									
**2-dose C−**	x			x			x		
**2-dose C+**	n.s	x		n.s	x		n.s	x	
**3-dose C−**	0.004	0.031	x	n.s	n.s	x	n.s	n.s	X
TP4									
**2-dose C−**	x			x			x		
**2-dose C+**	n.s	x		n.s	x		n.s	x	
**3-dose C−**	0.029	0.005	x	n.s	n.s	x	n.s	n.s	X
TP5									
**2-dose C−**	x			x			x		
**2-dose C+**	n.s	x		n.s	x		n.s	x	
**3-dose C−**	n.s	n.s	x	n.s	n.s	x	n.s	n.s	X

Immunization schematics were divided into the groups depending on the number of vaccination doses and COVID-19 history confirmed by the PCR test. Schemes are compared in pairs. The *p*-value was calculated with the non-parametric Mann–Whitney U test. *p* < 0.05 was considered statistically significant; 2 doses = two-dose scheme of BNT162b2 vaccination; 3 doses = three-dose scheme of BNT162b2 vaccination; C+ = positive SARS-CoV-2 PCR test; C− = negative SARS-CoV-2 PCR test; n.a = not available; n.s = not significant.

**Table A2:** Relationship of anti-SARS-CoV-2 spike protein Ab concentration (BAU/mL) after BNT162b2 vaccination at TP1–5, depending on the immunization scheme presented by the *p* value.

Scheme	2-Dose C+ TP2	2-Dose C+ TP3	2-Dose C+ TP4	2-Dose C−	3-Dose C− TP3	3-Dose C− TP4	2-Dose C+
TP1							
**2-dose C+ TP2**	x						
**2-dose C+ TP3**	n.a	x					
**2-dose C+ TP4**	n.a	n.a	x				
**2-dose C−**	n.a	n.a	n.s	x			
**3-dose C− TP3**	n.a	n.a	n.s	n.s	x		
**3-dose C− TP4**	n.a	n.a	n.s	0.002	n.s	x	
**2-dose C+**	n.a	n.a	0.02	0.003	0.003	<0.001	x
TP2							
**2-dose C+ TP2**	x						
**2-dose C+ TP3**	n.a	x					
**2-dose C+ TP4**	n.a	n.a	x				
**2-dose C−**	n.a	n.a	n.s	x			
**3-dose C− TP3**	n.a	n.a	n.s	0.02	x		
**3-dose C− TP4**	n.a	n.a	n.s	<0.001	n.s	x	
**2-dose C+**	n.a	n.a	0.02	0.04	0.01	<0.001	x
TP3							
**2-dose C+ TP2**	x						
**2-dose C+ TP3**	n.a	x					
**2-dose C+ TP4**	n.a	n.s	x				
**2-dose C−**	n.a	n.s	n.s	x			
**3-dose C− TP3**	n.a	n.s	n.s	0.003	x		
**3-dose C− TP4**	n.a	n.s	n.s	<0.001	n.s	x	
**2-dose C+**	n.a	n.s	n.s	n.s	0.006	0.009	x
TP4							
**2-dose C+ TP2**	x						
**2-dose C+ TP3**	n.a	x					
**2-dose C+ TP4**	n.a	n.s	x				
**2-dose C−**	n.a	0.03	n.s	x			
**3-dose C− TP3**	n.a	0.03	n.s	n.s	x		
**3-dose C− TP4**	n.a	0.02	n.s	<0.001	<0.001	x	
**2-dose C+**	n.a	n.s	0.006	n.s	n.s	<0.001	x
TP5							
**2-dose C+ TP2**	x						
**2-dose C+ TP3**	n.a	x					
**2-dose C+ TP4**	n.a	n.a	x				
**2-dose C−**	n.a	n.a	n.s	x			
**3-dose C−TP3**	n.a	n.a	n.s	n.s	x		
**3-dose C−TP4**	n.a	n.a	n.s	n.s	n.s	x	
**2-dose C+**	n.a	n.a	n.s	n.s	n.s	n.s	x
Delta S 3-5							
**2-dose C+TP2**	x						
**2-dose C+TP3**	n.a	x					
**2-dose C+TP4**	n.a	n.s	x				
**2-dose C−**	n.a	n.s	0.02	x			
**3-dose C−TP3**	n.a	n.s	n.s	n.s	x		
**3-dose C−TP4**	n.a	0.04	n.s	<0.001	0.05	x	
**2-dose C+**	n.a	n.s	n.s	n.s	n.s	n.s	x

Immunization schematics were divided into the groups depending on the number of vaccination doses and COVID-19 history confirmed by the PCR test. Scheme markings were introduced chronologically. In the two-dose scheme, there was one C− group (negative PCR test) and four C+ (positive PCR test). TP corresponded to the COVID-19 infection. In the three-dose scheme, there were two groups of C−. TP corresponded to the third dose obtaining. Schemes are compared in pairs. The *p*-value was calculated with the non-parametric Mann–Whitney U test. *p* < 0.05 was considered statistically significant: 2-doses C+ TP2 = subject obtained two doses of vaccine and then had SARS-CoV-2 infection at TP2; 2-dose C+ TP3 = subject obtained two doses of vaccine and then had SARS-CoV-2 infection at TP3; 2-dose C+ TP4 = subject obtained two doses of vaccine and then had SARS-CoV-2 infection at TP4; 3-dose C− TP3 = subject obtained subject obtained three doses of vaccine, with the third dose at TP3, and did not have previous SARS-CoV-2 infection; 3-dose C− TP4 = subject obtained three doses of vaccine, with the third dose at TP4, and did not have previous SARS-CoV-2 infection; 2-dose C+ = subject had SARS-CoV-2 infection before the two-dose scheme; 2-dose C− = subject obtained two doses of vaccine and did not have previous SARS-CoV-2 infection; delta S 3–5 = difference between anti-S1Ab titer at TP3 and TP5 after the second dose; n.a= not available; n.s = not significant.

**Table A3:** Comparison of Anti-SARS-CoV-2 spike protein Ab concentration (BAU/mL), after BNT162b2 vaccination, in all participants with different immunization schemes, depending on the number of vaccination doses and COVID-19 history presented by *p* value.

Scheme		2 Doses C+		2 Doses C−		3 Doses C−			
	*n* = 19	Median (Q1-Q3)	*n* = 44	Median (Q1-Q3)	*n* = 55	Median (Q1–Q3)	*p* value *	*p* value ^†^	*p* value ^‡^
TP1		1413.4 (784.6 to 3523.8)		220.3 (18.3 to 1371.8)		1.7 (0.0 to 21.1)	0.042	0.002	0.001
TP2		2469.3 (1284.8 to 4874.7)		2312.5 (1032.2 to 4422.5)		143.8 (11.4 to 632.5)	n.s	<0.001	<0.001
TP3		806.8 (483.0 to 1813.2)		665.5 (255.9 to 1703.0)		92.0 (4.4 to 281.6)	n.s	<0.001	0.001
TP4		619.3 (317.6 to 2634.1)		368.9 (154.1 to 1338.0)		64.7 (15.6 to 470.7)	n.s	0.005	0.005
TP5		754.6 (466.4 to 1922.5)		382.1 (200.5 to 2852.6)		407.7 (84.5 to 1619.2)	n.s	n.s	n.s
Delta S3-5		−517.0 (−1084.5 to 9.7)		−316.0 (−538.2 to −130.5)		216.0 (31.2 to 3867.0)	n.s	<0.001	n.s

BNT162b2 vaccination effect (Ab titer) in all participants, depending on the number of vaccination doses and COVID-19 history confirmed by a positive PCR test. Values are set as the median (MD) and quartile interval (QR1–QR3); the *p*-value was calculated with the non-parametric Mann–Whitney U test, and *p* < 0.05 was considered statistically significant; * is the difference between Ab titer in two-dose C+ and two-dose C− schemes; ^†^ is the difference between Ab titer in two-dose C− and three-dose C− schemes; ^‡^ is the difference between Ab titer in two-dose C+ and three-dose C− schemes. Delta S 3–5 = difference between anti-S1Ab titer at TP3 and TP5 after the second dose; 2 doses = two-dose scheme of BNT162b2 vaccination; 3 doses = three-dose scheme of BNT162b2 vaccination; C+ = positive SARS-CoV-2 PCR test; C− = negative SARS-CoV-2 PCR test; *n* = number of observations; n.s = not significant.
